# RAP1 GTPase Overexpression is Associated with Cervical Intraepithelial Neoplasia

**DOI:** 10.1371/journal.pone.0123531

**Published:** 2015-04-09

**Authors:** Marcelo Antonio Pascoal-Xavier, Anna Carolina Cançado Figueiredo, Luciana Inácia Gomes, Vanessa Peruhype-Magalhães, Carlos Eduardo Calzavara-Silva, Marcelo Azevedo Costa, Ilka Afonso Reis, Claudio Antônio Bonjardim, Erna Geessien Kroon, Jaquelline G. de Oliveira, Paulo César Peregrino Ferreira

**Affiliations:** 1 Departamento de Microbiologia, Instituto de Ciências Biológicas, Universidade Federal de Minas Gerais, Belo Horizonte, Minas Gerais, Brasil; 2 Centro de Pesquisas René Rachou/FIOCRUZ, Belo Horizonte Minas Gerais, Brasil; 3 Departamento de Engenharia de Produção, Escola de Engenharia, Universidade Federal de Minas Gerais, Belo Horizonte, Minas Gerais, Brasil; 4 Departamento de Estatística, Instituto de Ciências Exatas, Universidade Federal de Minas Gerais, Belo Horizonte, Minas Gerais, Brasil; University of Navarra, SPAIN

## Abstract

RAP1 (RAS proximate 1), a small GTP-binding protein of the *RAS* superfamily, is a putative oncogene that is highly expressed in several malignant cell lines and types of cancers, including some types of squamous cell carcinoma. However, the participation of RAP1 in cervical carcinogenesis is unknown. We conducted a cross-sectional study of paraffin-embedded cervical biopsies to determine the association of RAP1 with cervical intraepithelial neoplasia (CIN). Standard and quantitative immunohistochemistry assessment of RAP1 expression in fixed tissue was performed on 183 paraffin-embedded cervical biopsies that were classified as normal or non-dysplastic mucosa (NDM) (n = 33); CIN grade 1 (n = 84) and CIN grade 2/3 (n = 66). A gradual increase in RAP1 expression in NDM < CIN 1 < CIN 2/3 (*p<0*.*001*) specimens was observed and was in agreement with the histopathologic diagnosis. A progressive increase in the RAP1 expression levels increased the risk of CIN 1 [odds ratio (OR) = 3.50; 95% confidence interval (CI) 1.30-10.64] 3.5 fold and the risk of CIN 2/3 (OR = 19.86, 95% CI 6.40-70.79) nearly 20 fold when compared to NDM. In addition, stereotype ordinal regression analysis showed that this progressive increase in RAP1 expression more strongly impacted CIN 2/3 than CIN 1. Our findings suggest that RAP1 may be a useful biomarker for the diagnosis of CIN.

## Introduction

Cervical cancer, which is caused by high-risk *Human papillomavirus* (HR-HPV), is the second most frequent cancer in women worldwide [1]. Great efforts have been made during the last two decades to obtain useful markers that can be used in clinical practice for the proper identification and follow-up of precursor lesions of cervical cancer, also known as cervical intraepithelial neoplasia (CIN). Currently, the main biomarkers applied for CIN detection are proteins related to the cell cycle, such as p16^INK4A^, Ki-67, minichromosome maintenance 7 and 2 (MCM7 and 2), topoisomarase II alpha, and cyclin D1, all of which have altered expression due to the effects of HPV on the cell cycle. The p16^INK4A^ protein the best known surrogate biomarker of high grade lesions and also considered an indicator of the E6 and E7 overexpression in cervical lesions [2–5]. Among the small GTPases, current research indicates the participation of RAC1 and RHO in the progression of cervical neoplasia [6,7]. However, the participation of RAP1 GTPase in cervical carcinogenesis remains unknown.

RAP1 (RAS proximate 1), a small GTPase of the RAS superfamily, and its regulatory proteins are involved in cell cycle progression, differentiation, survival and adhesion [8,9]. RAP1 is activated by a wide variety of external stimuli, and it functions as a signal transducer that switches between its inactive GDP-bound form and its active GTP-bound form. This switch is regulated by several guanine nucleotide exchanger factors (GEFs), which serve as activators, and GTPase-activating proteins (RAPGAPs), which act as inactivators [10,11]. RAP1 seems to play a role in cancer cell growth, invasion, and metastasis, and dysregulation of RAP1 activation has been described in several malignant cell lines and cancers, such as oropharyngeal squamous cell carcinoma (SCC), papillary thyroid cancer, breast cancer, renal cell carcinoma, and melanoma [12–18].

Associations between HPV-mediated oncogenesis and alteration of the RAP1 signaling pathway have been described. The HR-HPV E6 oncoprotein targets the protein E6TP1, a known RAPGAP, for proteasome-dependent degradation and consequently enhances the GTP loading of RAP1 with subsequent activation of the MAPK signaling pathway and RAP1 overexpression [19,20]. Likewise, in HPV-infected epithelial cells, the interaction of the HPV E2 protein with the cellular bromodomain protein Brd4, a cell cycle progression regulator, enhances the RAP GAP activity of SPA-1, which disrupts the proper balance of RAP1 activation [21].

Those findings led us to investigate the expression levels of RAP1 in low- and high-grade CIN lesions to address the potential use of this putative oncogene as a cervical neoplasia biomarker. By comparing the immunostaining pattern of RAP1 in cervical specimens of CIN 1 and CIN 2/3 to that of non-dysplastic mucosa (NDM), we verified that RAP1 expression gradually increased with the severity of the cervical lesions. Moreover, we found a strong association of RAP1 overexpression in high grade lesions.

## Materials and Methods

### Ethics Statement

This cross-sectional study was approved by the institutional review board of the Universidade Federal de Minas Gerais (certificate number CAAE-0397.0.203.245–09). The need for written informed consent from the donor or the next of kin for the use of the paraffin-embedded samples in this research was waived by the institutional review board of the Universidade Federal de Minas Gerais.

### Study Participants

**Fig 1:**
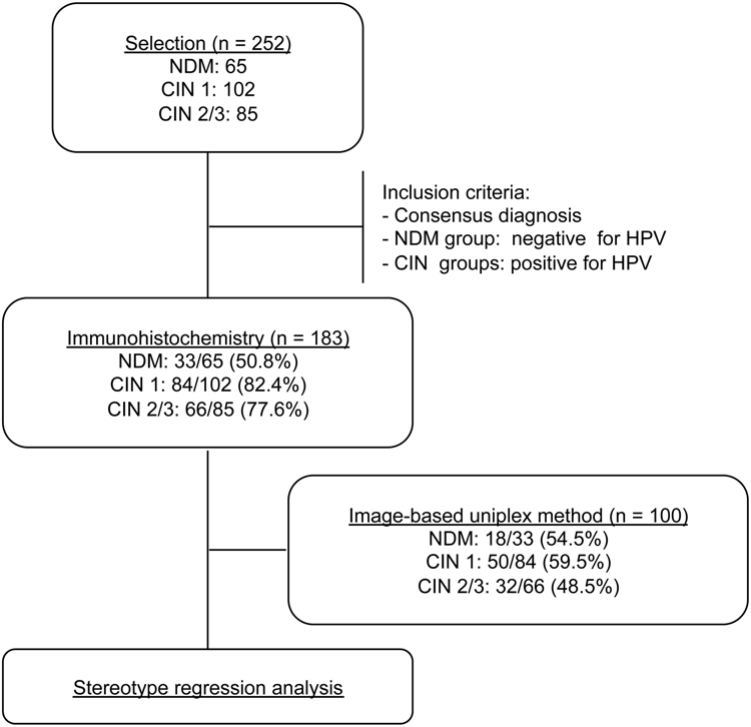
Study procedures and outcomes. The flow diagram shows the number of samples that were obtained from the laboratory file as well as those that were included in the immunohistochemistry and statistical analyses.

Two hundred and fifty-two paraffin-embedded cervical biopsies, normal or NDM (n = 65), CIN 1 (n = 102) and CIN 2/3 (n = 85), were collected from different individuals between May 2009 and March 2011 from the archives of the Hospital das Clínicas, Universidade Federal de Minas Gerais, Brazil. The majority of the patients were predominantly from the State of Minas Gerais, Brazil. Patients from other states, of the Southeast, North and Northeast regions of Brazil were also included. The mean age of the patients was 38 years, ranging from 18 to 74 years old. Inclusion criteria were reviewed and classified as consensus diagnosis by two certified pathologists (M.A.P.X. and L.P.F.) and HPV DNA detection. The study procedures are summarized in the flow diagram shown in Fig 1.

### HPV genotyping

DNA was extracted from five serial sections (10 μm) of each paraffin-embedded sample using the QIAamp DNA FFPE Tissue Kit (Qiagen, Düsseldorf, Germany) according to the manufacturer's instructions. Nested PCR amplification was performed using the external primers MY11 and MY09 and the internal primers GP5+ and GP6+, which were designed to amplify a 150-bp product from the L1 gene of several HPV types, as described elsewhere [22,23]. For HPV genotyping, the gel-purified, 150-bp amplicons (QIAquick Gel Extraction Kit, QIAGEN Inc., CA, USA) were sequenced in both directions (Applied Biosystems 3730 DNA Sequencer; Life Technologies, USA), and a BLAST search was carried out to identify the HPV types. DNA extracted from HPV-18-infected HeLa cells was used as a positive control. To determine the DNA integrity and identify the presence of PCR inhibitors, all samples were also tested by PCR using primers PC03 and PC04, which amplified the beta-actin gene [24].

### Immunohistochemistry

RAP1 and p16^INK4A^ immunostaining was performed on 4-μm sequential sections of formalin-fixed, paraffin-embedded biopsy specimens using the Novolink Polymer Detection System (Novocastra, Leica Microsystems, USA). Deparaffinization with xylene and rehydration through a graded alcohol series was followed by antigen retrieval by heating to 90°C for 10 minutes in EDTA buffer, pH 9.0. After blocking endogenous peroxidases (DakoCytomation, Glostrup, Denmark), the deparaffinized sections were incubated for 1 hour at room temperature with anti-RAP1 or anti-p16^INK4A^ antibodies (rabbit polyclonal to RAP1A+RAP1B; ab40814, Abcam, 1:120 dilution; mouse monoclonal to p16^INK4A^, 551154; clone G175-405, BD Pharmingen Bioscienses, dilution 1:110). The sections were then incubated at room temperature for 30 minutes with the Novolink polymer DS and washed twice with Tris-borate saline for 5 minutes. Peroxidase activity was developed using a diaminobenzidine (DakoCytomation, Glostrup, Denmark) solution for 30 minutes at room temperature, counterstaining was performed using Harris hematoxylin, and then evaluated by light microscopy.

RAP1 and p16^INK4A^ expression levels in the NDM and CIN groups were evaluated according to criteria previously described by Galgano and collaborators [25]. The scoring of RAP1 and p16^INK4A^ was graded as pattern 0 (no staining), pattern 1 (weak staining in 5%-25% of the cells distributed in the basal third of the epithelium), pattern 2 (moderate staining in 26%-50% of the cells distributed in the basal third of the epithelial thickness), and pattern 3 (strong staining in >50% of the cells that were distributed in two-thirds of the entire epithelial thickness). RAP1 and p16^INK4A^ expression levels were considered normal for pattern 0 or 1 and high for pattern 2 or 3.

To limit the potentially subjective visual assessment of the RAP1 and p16^INK4A^ immunohistochemical score, we used the image-based uniplex method [16] to measure the relative intensity (RI) of RAP1 and p16^INK4A^ protein expression in a representative number of samples. The 16-bpp TIF images of the tissue were captured using an AxioCam MRc digital camera equipped with AxioVision software and attached to a Zeiss Axio microscope (Carl Zeiss Microscopy, Cambridge, UK). Images spanning the full range of intensity levels were analyzed using ImageJ 1.37v processing software (National Institutes of Health), which provided area and pixel value statistics of the user-selected region of interest (ROI). Ten 1-mm^2^ areas were selected as ROIs in the epithelium and stroma by a certified pathologist (M.A.P.X.). The RI of biomarker staining (relative protein expression) was calculated in RI units by dividing the mean pixel intensity of equal ROIs containing stroma (*I*background) by the mean pixel intensity of equal ROIs containing epithelial cells (*I*test). This algorithm accounted for variations in background staining related to the immunohistochemistry routine.

### Statistical analysis

Associations between categorical variables were evaluated using Fisher's exact test, and differences between continuous variables were determined using the two-sample T-test. To estimate odds ratios (OR) and the corresponding 95% confidence intervals (CIs) for the association between CIN and change in RAP1 expression, we compared normal and high expression of NDM to that of CIN 1 and of CIN 2/3 and CIN 1 to that of CIN 2/3. To establish a relationship between CIN and the change in RAP1 expression, we fitted the stereotype ordinal logistic regression model, $log\left[\frac{\pi_{j}}{\pi_{NDM}}\right]\;=\;\alpha_{j}+\;\emptyset_{j}\beta x$, where *j* = 2,3 represents the CIN 1 and CIN 2/3 classes, respectively, and x = 0,1,2,3 represents the RAP1 immunohistochemical expression, and we estimated the value of *β* as proposed by Anderson [26]. The coefficient significance was tested using the Log-likelihood method. A result was considered statistically significant when p<0.05. The statistical analyses were performed using MINITAB 16 (Minitab Inc., State College, Pennsylvania) and R (Version 2.15.3; http://www.r-project.org) with the VGAM package statistical softwares.

## Results

### RAP1 expression and HPV detection

**Fig 2:**
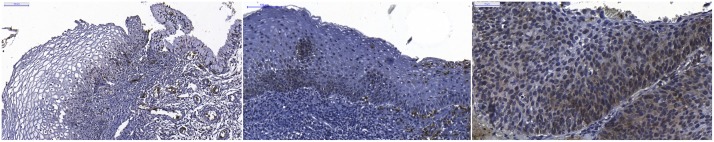
Immunohistochemical staining of RAP1. Left—Squamocolumnar junction of non-dysplastic cervical mucosa (NDM) showing very weak immunostaining for RAP1 in the cytoplasm of basal cells and no staining in the intermediate and superficial cells. Middle—CIN 1 showing moderate staining for RAP1 in cells distributed in the basal third of the epithelium and showing RAP1 expression in neutrophils. Right- CIN 2/3 showing strong staining for RAP1 in cells diffusely distributed throughout the entire epithelial thickness, with nuclear translocation of RAP1.

The RAP1 and p16^INK4A^ expression levels in NDM, CIN 1, and CIN 2/3 lesions are shown in Fig 2, in S1 Fig and Table 1, which also include the HPV-16, HPV-18 and HPV-31 types that were detected in the CIN groups. p16^INK4A^ was used as an immunohistochemical reference for our study. RAP1 was undetectable in 36.4% of the NDM samples (12/33), and once verified in the NDM group, RAP1 immunostaining was very weak and predominantly restricted to the basal layer of the epithelium in 42.4% of the samples (14/33) and was classified as pattern 1 (as described in Material and Methods). Only 7 of the 33 cases of NDM presented moderate staining of RAP1 in 26%-50% of the cells that were distributed in the basal third of the epithelial thickness (pattern 2). Unlike what was observed in NDM, RAP1 expression was observed in 100% of the CIN 1 samples. About half of the CIN 1 samples (43/84) presented a moderate RAP1 immunostaining in dysplastic cells in the basal third of the epithelium (pattern 2), and the other half presented weak immunostaining of RAP1 expression, as described for pattern 1. In contrast, 85% (56/66) of the CIN 2/3 lesions presented moderate or strong immunostaining, indicating much higher RAP1 expression levels in high-grade lesions than those observed in NDM or CIN 1 biopsies (*p*<0.001). Indeed, a predominance of strong, diffuse RAP1 staining throughout the thickness of the epithelium was observed in 59.1% (39/66) of the CIN 2/3 lesions. Notably, p16^INK4A^ showed the same immunohistochemical patterns described by Galgano and colleagues in NDM, CIN1 and CIN2/3 samples [25]. Strong nuclear staining for RAP1 was observed in approximately 70% of the CIN 2/3 lesions (Fig 2C), while weak nuclear staining was only observed in some basal cells of the CIN 1 (Fig 2B and 2A). No cytoplasmic or nuclear staining was observed in intermediate or superficial cells of the NDM samples (Fig 2A). RAP1 was strongly expressed in neutrophils and endothelial cells and was weakly expressed in lymphocytes and stromal mononuclear cells (Fig 2B and 2C).

**Table 1:** Scores of RAP1 and p16^INK4A^ immunohistochemical expression in precursor lesions of cervical neoplasia.

Immunohistochemistry	NDM	CIN 1	CIN 2/3
**RAP1**	**N (%)**	**N (%)**	**N (%)**
0	12 (36.4)	0 (0)	6(9.1)
1	14 (42.4)	43 (51.2)	4 (6.1)
2	7 (21.2)	41 (48.8)	17 (25.8)
3	0 (0)	0 (0)	39 (59.1)
Total	33 (100)	84 (100)	66 (100)
**p16^INK4A^**	**N (%)**	**N (%)**	**N (%)**
0	11 (33.4)	3 (3.6)	2 (3.0)
1	14 (42.4)	33 (39.3)	2 (3.0)
2	8 (24.2)	47 (56.0)	3 (4.5)
3	0 (0)	1 (1.2)	59 (89.4)
Total	33 (100)	84 (100)	66 (100)
**Level expression**	**NDM**	**CIN 1**	**CIN 2/3**
**RAP1**	**N = 33**	**N = 84**	**N = 66**
Normal (pattern 0 or 1)	26	43	10
High (pattern 2 or 3)	7	41	56
**p16^INK4A^**	**N = 33**	**N = 84**	**N = 66**
Normal (pattern 0 or 1)	25	36	4
High (pattern 2 or 3)	8	48	62
**Presence of cervical**	**NDM**	**CIN 1**	**CIN 2/3**
**High-risk HPV DNA**	**N (%)**	**N (%)**	**N (%)**
HPV-16	0 (0)	51 (60.7)	48 (72.7)
HPV-18	0 (0)	32 (38.1)	17 (25.8)
HPV-31	0 (0)	1 (1.2)	1 (1.5)

Abbreviations: NDM—non-dysplastic mucosa; CIN—cervical intraepithelial neoplasia.

A predominance of HPV-16 in 60.7% (51/84) and 72.7% (48/66) of the CIN 1 and CIN 2/3 samples, respectively, was observed. HPV-18 was detected in 38.1% (32/84) and 25.8% (17/66) of the CIN 1 and CIN 2/3 samples, respectively. HPV-31 was detected in only one sample from each group (Table 1).

### Morphometric evaluation of RAP1 expression

**Fig 3:**
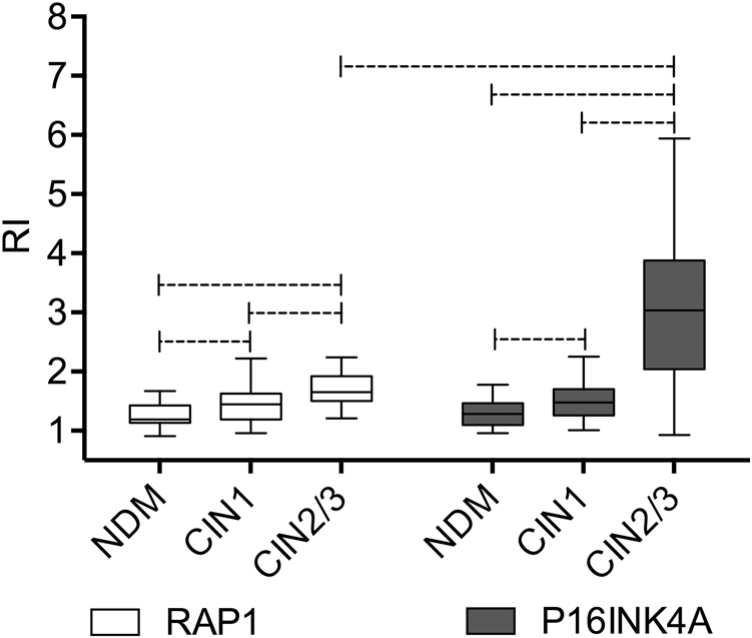
Morphometric analysis of RAP1 and p16^INK4A^ immunohistochemical staining. Relative Intensity (RI) data of RAP1 anfd p16^INK4A^ expression in NDM, CIN 1, and CIN 2/3 samples are expressed in box plot format, with boxes stretching from the 25^th^ percentile to the 75^th^ percentile and the line across the box representing the median values. Statistically significant differences (p <0.05) are shown by dotted lines.

To limit the subjective visual assessment of immunohistochemical staining, we also performed a morphometric evaluation of RAP1 and p16^INK4A^ expression levels. We measured the relative intensity (RI) of chromogen staining as a measure of the relative RAP1 and p16^INK4A^ protein expression in NDM, CIN 1, and CIN 2/3 lesions (Fig 3). Our data showed higher RAP1 expression in CIN 2/3 samples compared to NDM and CIN 1 samples (*p*<0.05) and in CIN 1 samples compared to NDM samples (*p*<0.05), similar to the data obtained by standard microscopy analyses. RAP1 and p16^INK4A^ immunohistochemical morphometric patterns were similar except for the significant difference (p<0.05) between RAP1 and p16^INK4A^ expression observed in the CIN2/3 group (Fig 3).

### Overexpression of RAP1 and CIN progression

The association between RAP1 expression and risk of CIN progression was determined to perform comparisons between all groups. The table 2 presents the odds ratios (ORs) and 95% confidence intervals (CIs) for the study of association between RAP1 expression levels (normal and high) in the NDM, CIN 1, and CIN 2/3 samples. We observed that high levels of RAP1 raised the risk of CIN 1 by 3.5 fold [(OR) = 3.50; 95% confidence interval (CI) 1.30–10.64] and the risk of CIN 2/3 by nearly 20 fold (OR  =  19.86, 95% CI 6.40–70,80) compared to NDM and increased the risk of CIN 2/3 5.8 fold (OR = 5.80; 95% CI 2.51–14.52) compared to CIN 1.

**Table 2:** Associations between RAP1 expression and risk of CIN progression.

Ratio	OR	95% CI	*p*-value
CIN 1 / NDM	3.50	1.30,10.64	<0.001
CIN 2/3 / NDM	19.87	6.40,70.80	<0.001
CIN 2/3 / CIN 1	5.80	2.51,14.52	<0.001

Abbreviations: OR—Odds ratio; NDM—non-dysplastic mucosa; CIN—cervical intraepithelial neoplasia.

The relationship between CIN grade and variation in RAP1 expression were determined using stereotype regression analysis. The table 3 summarizes the results of the fitted model. According to the model, $log\left[\frac{\pi_{j}}{\pi_{NDM}}\right]\;=\;\alpha_{j}+\;\emptyset_{j}\beta x$, *j* = 2,3 and x = 0,1,2,3 and with identifiability constraints (∅_CIN 2/3_ = 1, ∅_NDM_ = 0) as in Anderson and colleagues (26), we estimate ∅_CIN 2/3_ = 0.36 and β = 2.98. Therefore, we estimated the relation between risks ratio and RAP1 expression is $log\left[\frac{\pi_{CIN\;2/3}}{\pi_{NDM}}\right]\;=\;-4.56+2.98x$ for CIN2/3 and $log\left[\frac{\pi_{CIN\;1}}{\pi_{NDM}}\right]\;=\;-0.30+\left(0.36\right)2.98x$ for CIN1. Beta value is statistically different from 1, showing the influence of the increasing in RAP1 in the risk of CIN compared to NDM.

**Table 3:** Estimated coefficients, standard errors, z-scores, and two-tailed *p*-values for the fitted stereotype regression model.

Coefficients	Estimate (95% CI)	SE	z-score	*p* value
∅NDM	0*	—	—	—
∅CIN 1	0.36 (0.22,0.50)	0.07	4.73	<0.001
∅CIN 2/3	1*	—	—	—
αCIN 1	-0.30 (-1.04,0.44)	0.38	-0.79	0.2146
αCIN 2/3	-4.56 (-6.05,-3.07)	0.76	-5.97	<0.001
β	2.98 (2.16,3.80)	0.42	7.10	<0.001

Log-likelihood: -136.46

Abbreviations: SE—Standard error; NDM—non-dysplastic mucosa; CIN—cervical intraepithelial neoplasia.

* Identifiability constraints proposed proposed by Anderson [26].

However, since the estimate for the weight of the RAP1 expression influence in risks ratios for CIN1 class, ∅_CIN 1_, is statistically smaller than 1, stereotype ordinal regression analysis also showed that this progressive increase of RAP1 impacts more strongly in CIN2/3 than CIN1. Deviance residuals of the model indicated general goodness of fit of the model to the obtained data.

## Discussion

In this cross-sectional study, we found a strong association of RAP1 with a significant increased risk of progression of CIN 1 and CIN 2/3 by comparing the RAP1 protein expression levels at different degrees of cervical intraepithelial neoplasia using standard and semi-quantitative approaches. RAP1 expression levels were predominantly undetectable or basal in normal squamous mucosa, but RAP1 expression gradually increased according to the severity of the cervical lesions.

The two main limitations of this study were the quality of the DNA that was extracted from paraffin-embedded samples for HPV detection and typing and the subjective bias of the qualitative assessment of immunohistochemistry. To circumvent those limitations, we used a large number of cervical lesions and performed morphometric analysis, respectively. Another limitation was the interference of nonepithelial cell immunostaining in the RAP1 morphometric analyses. The constitutive expression of RAP1 in neutrophils, lymphocytes and endothelial cells may have interfered with the calculation of the relative intensity (RI) index due to the increased number of inflammatory cells and angiogenesis that was frequently observed during CIN progression [27].

A very weak RAP1 cytoplasmic expression was observed in the basal cells of some NDM samples presenting inflammatory infiltrate in its the stromal tissue (Fig 2A). This may be due to cell proliferation induced by the inflammatory process of the cervical mucosa. Interestingly, a very discrete immunostaining of p16^INK4A^ was also observed in some NDM inflammatory cervical samples (S1 Fig) as also described elsewhere [28].

We observed a strong nuclear staining for RAP1 in CIN 2/3 samples, as previously reported in oropharyngeal SCC cell lines and human oral cancer specimens, and for other small GTPases, such as RHO and RAC1, in premalignant lesions and cervical cancer [6,7,12,29]. Because only the active GTP-bound form of RAP1 translocates to the nucleus, we can infer that the strong nuclear staining of RAP1 that was observed in the CIN 2/3 specimens indicated the presence of active RAP1 and its putative role in the malignant process [11,12].

In this context, RAP1 is a cellular biomarker because it can distinguish different groups of precursor lesions of cervical carcinoma [2,25]. As shown in Table 3, our analyses support the use of RAP1 as a CIN biomarker because both the coefficient α_CIN 1_ and coefficient α_CIN 2/3_ influence the *β* value. However, the weight of the growing shift in the immunohistochemical pattern of logit $log\left[\frac{\pi_{CIN\;2/3}}{\pi_{NDM}}\right]$ is much more relevant than in the logit $log\left[\frac{\pi_{CIN\;1}}{\pi_{NDM}}\right]$. For such risk estimates, we use the parsimonious stereotype model, which is more flexible and better preserves the ordered outcomes by imposing a linear structure in the model’s logit [30,31]. In addition to its use as a diagnostic and potential risk progression biomarker, an advantage of RAP1 could be the manipulation of RAP1 signaling pathway components in the cervical microenvironment [32].

The very different expression patterns of RAP1, i.e., weak/moderate expression in CIN 1 lesions in contrast to the strong RAP1 expression observed in the vast majority of CIN 2/3 lesions, suggest a distinct influence of HPV on RAP1 expression during the development of cervical carcinogenesis. CIN 1 and epithelial mucosa persistently infected with HPV are characterized by high levels of cytoplasmic and nuclear HPV E2 protein, which binds to the Brd4 protein, a cellular protein that mediates several processes important for the viral life cycle, including viral genome maintenance and replication [33,34]. When bound to Brd4, E2 competes with SPA-1, a RAPGAP. Hence, it is possible that E2-Brd4 binding favors the dysfunction of SPA-1, creating a hyperactive state of RAP1 [35]. On the other hand, overexpression of HR-HPV E6 protein, especially after integration of the HPV genome into the host genome in high-grade dysplasia (CIN 2/3), may activate RAP1-mediated signaling pathways during cellular transformation through the down-regulation of E6TP1 [36].

Our results suggest that RAP1 GTPase acts as a new biomarker with potential clinical value for severity assessment of cervical epithelial neoplasia. However, studies of RAP1 expression and its regulators in epithelial cells that are associated with HPV infection as well as prospective observational studies with a long-term follow-up will be required to elucidate the role of RAP1 in cervical carcinogenesis and to identify the clinical relevance of RAP1 in cervical cancer progression.

## Supporting Information

S1 Fig Immunohistochemical staining of p16^INK4A^. Left—Squamocolumnar junction of non-dysplastic cervical mucosa (NDM) showing very weak immunostaining for p16^INK4A^ in the cytoplasm of basal cells and no staining in the intermediate and superficial cells. Middle—CIN 1 showing moderate staining for p16^INK4A^ in cells distributed in the basal third of the epithelium. Right—CIN 2/3 showing very strong staining for p16^INK4A^ in cells diffusely distributed throughout the entire epithelial thickness.
(TIF)

S1 Dataset Data set for Fig 3 “Morphometric analysis of RAP1 and p16^INK4A^ immunohistochemical staining.” (XLSX)

S2 Dataset Dataset for Table 1 “Scores of RAP1 and p16^INK4A^ immunohistochemical expression in precursor lesions of cervical neoplasia”. (XLSX)

S3 Dataset Dataset for Table 2 “Associations between RAP1 expression and risk of CIN progression”. (XLSX)

S4 Dataset Dataset for Table 3 “Estimated coefficients, standard errors, z-scores, and two-tailed *p*-values for the fitted stereotype regression model”. (XLSX)
