# Supplementary figures and images for: RAP1 GTPase Overexpression is Associated with Cervical Intraepithelial Neoplasia
Source: PLoS One. 2015 Apr 9;10(4):e0123531. doi: 10.1371/journal.pone.0123531 (PMC4391937; doi:10.1371/journal.pone.0123531)

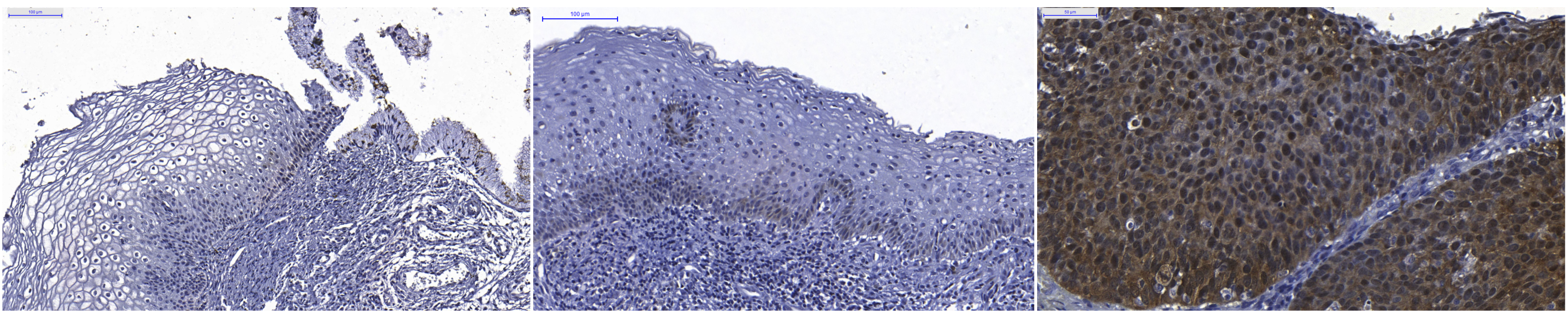

Supplement: S1 Fig — Left—Squamocolumnar junction of non-dysplastic cervical mucosa (NDM) showing very weak immunostaining for p16INK4A in the cytoplasm of basal cells and no staining in the intermediate and superficial cells. Middle—CIN 1 showing moderate staining for p16INK4A in cells distributed in the basal third of the epithelium. Right—CIN 2/3 showing very strong staining for p16INK4A in cells diffusely distributed throughout the entire epithelial thickness. (TIF) [file pone.0123531.s001.tif]
